# Supplementary material for: Are carbon emissions trading and green financial instruments synergistic? -Comprehensive quantitative research based on content analysis
Source: PLoS One. 2024 Mar 7;19(3):e0298601. doi: 10.1371/journal.pone.0298601 (PMC10919601; doi:10.1371/journal.pone.0298601)
Supplement: S1 Data — (ZIP) [file pone.0298601.s001.zip › Data/地级市Z_F工作报告.zip!/╝¬┴╓/░╫│╟╩╨/└┤╫╘╣1⁄2╓┌║┼í╛┬φ┐╦╩2╛▌═°í┐_2004.docx]

各位代表：

　　白城市第二届人民政府任期已满。现在，我代表市第二届人民政府，向市第三届人民代表大会第一次会议报告工作，请予审议，并请市政协各位委员提出意见。

　　一、过去五年经济社会发展的回顾

　　1999年以来，在市委领导下，在市人大依法监督和市政协民主监督下，我们认真贯彻“三个代表”重要思想，围绕全面实施“三步走、三基本”战略规划，团结和带领全市广大干部群众抢抓机遇，开拓进取，奋发图强，把白城推向了大开发、大建设、大发展的新时期。过去的五年，是全市上下坚持与时俱进推进思想大解放的五年，是国民经济和社会事业持续快速发展的五年，是白城面貌发生历史性变化的五年。

　　——综合经济实力显著增强。2001年、2002年我市在全省社会经济综合实力排序中连续升两位，各县（市）普遍升了2-8位。2003年全市国内生产总值完成123.2亿元，比1998年增长95.3%，年均增长14.3%，其中第一产业增加值43.5亿元，比1998年增长91.7%，年均增长13.9%；第二产业增加值40.9亿元，比1998年增长117.9%，年均增长16.9%；第三产业增加值38.8亿元，比1998年增长79%，年均增长12.3%。固定资产投资完成46.2亿元，比1998年增长418.5%，年均增长39%。全口径财政收入完成98390万元，比1998年增长106%，年均增长15.5%。

　　围绕兴工富市，推进企业改革、重组和技术进步，着力提高运行质量，工业速度、效益实现快速增长。2000年全市工业实现了整体扭亏为盈，经济效益逐年提高。2003年规模以上工业产值完成46亿元，年均增长15.2%；实现利润1.92亿元，年均增长24%。五年完成技术改造243项，投资23.8亿元，年均增长22.8%。主导产业形成了新的发展格局，汽车配套、纺织服装、医药、农产品加工等重点行业实现利税占规模以上工业的80%。骨干企业群体和规模不断发展壮大，白城派克公司、纺织公司、通业公司等重点企业实现产值占规模以上工业的30%。重点产品基本实现了技术性开发、集约化生产、品牌化经营，市场竞争力进一步提高。

　　立足增加农民收入，调整优化结构，转变农业增长方式，农村经济全面发展。2003年农业产值完成80.9亿元，年均增长15.9%。万元田棚发展到13.6万户，股份牧业户发展到1.2万户。落实建设各类农业园区117个，其中中国吉林科尔沁精品牧业发展区、镇赉百万只羔羊产业化园区、洮南百万亩山杏园区、通榆中国草原红牛园区、大安农业科技示范园区等一批园区已初具规模。建成各类农产品加工企业731个，农村中介服务组织发展到375个。加强生态建设，五年完成水利综合工程量2790万立方米，节水灌溉面积340万亩；科学利用水资源，去年汛期月亮湖水库等蓄水10亿立方米；在全省率先建成数字化立体大气探测网络，实现了气象装备现代化，人工影响天气能力不断增强；完成造林278.8万亩，营造生态草138.5万亩，草原围栏238万亩，森林覆盖率由1998年的9.8%提高到12.5%。加快蚕业开发，植桑2.5万亩，实现了植桑养蚕的突破。

　　大力发展商贸经济，不断拓展国内外市场，流通领域繁荣活跃。2003年社会消费品零售总额完成56.6亿元，年均增长8.4%；外贸进出口额完成4530万美元，年均增长18.8%；开发了吉鹤、鹤城等品牌系列卷烟，累计销售2.3万大箱；粮食仓储设施进一步完善，销售粮食连续超额完成省下达计划。市场建设步伐加快，建设了新世纪购物中心、阳光广场、聚龙建材城、瑞光商贸城、洮南杂粮杂豆、大安白鹅、镇赉大牲畜、通榆关东马市、洮北红干椒等专业市场45个，其中万米以上市场24个。建成了6条步行街，超市、连锁、仓储、分销、代理等现代流通业迅速发展。

　　坚持以项目为载体，加大了工作力度，项目建设步伐加快。五年完成投资151亿元，建设了派克高压阻尼点火线总成、百万亩治沙工程、农村电网改造等130个重点项目。石油、风电开发取得新进展，2003年我市境内采油厂石油产量153万吨。建成了通榆同发风电厂，累计发电量2.9亿千瓦；洮北青山、洮南大通、通榆团结等三个风电项目已正式签约。霍白铁路、白城第二火力发电厂等一批重大项目取得了阶段性进展。抓住振兴东北老工业基地的战略机遇，重新调整规划了投资超千万元项目180项，其中投资超亿元项目109项，有70个投资亿元以上的项目被列入全省老工业基地调整改造规划，一批重点项目已通过专家论证，项目规划和建设水平有了新提高。

　　——改革开放实现新突破。坚持以体制和机制创新为重点，推进国有企业改革。全市96.1%的国有工商企业实现了改制，在全省率先实现了基本完成国有企业改制任务、基本消灭停产半停产企业、基本消除亏损大户的阶段性目标。精心组织，分类指导，规范操作，企业“双退”重组取得明显成效。非公有制经济快速发展2003年全市个体私营企业发展到8.7万户；实现增加值18.4亿元，实缴税金1.35亿元，年均分别增长22.9%和18.1%。完成了市县乡机构改革，积极探索事业单位改革。农村税费、粮食流通体制、土地使用权流转、科技教育卫生等改革全面推进。小城镇综合改革取得新成绩，岭下、瞻榆等10个镇进入省综合改革发展百强镇，安广镇被列为国家综合改革试点镇。

　　全方位扩大对外开放，大力招商引资、招贤引智。五年到位资金135.5亿元；引进各类人才670人，引进智力130项。美国德尔福、山东华金、四川希望、深圳宝安、武汉马应龙、吉林敖东等182户域外企业来我市投资兴业。经贸交流活动扩展到全国大部分沿海发达省、市，延伸到世界40多个国家和地区。组织群众走出去，发展劳务经济，五年劳务输出126万人（次），实现收入33.7亿元。开发区建设步伐加快，白城经济开发区、查干浩特旅游经济开发区、民营经济开发区等6个开发区（园区）累计完成固定资产投资24.2亿元，开发建设211个项目，GDP年均增长38%，增强了对全市经济的拉动力。

　　积极发展生态旅游业，以旅游开发促进开放、促进发展。五年投入7亿元，建设和完善了向海、莫莫格、查干浩特等156个景区景点。连续举办了中国?白城生态旅游节，组织开展了丰富多彩的旅游经贸活动，累计接待国内外游客200万人（次），旅游收入7.5亿元。

　　——城镇面貌发生巨大变化。按照“一城崛起、县城争辉、中心镇突破”的发展思路，运用市场机制经营城市，连续开展了城市开发建设管理总体战。五年投入资金126亿元，实施1200项工程，其中市区投资80亿元，实施728项工程。县城和中心镇面貌也发生了显著变化。全市新建、改造、拓宽道路469条，铺装面积430万平方米；新建住宅391万平方米；城市绿化覆盖率由1998年的23%提高到36%。建设了白城火车站、二环路、三水厂、西部供热站、客运大厦、大安新世纪广场、洮南市区广场、镇赉南湖生态园、通榆体育公园等一批城市设施。实施了亮化工程，亮化档次和水平进一步提高。蓝天工程取得成效，市区和县城大气环境质量分别达到了国家二级和三级标准，居全省前列。建成了92个较高标准的社区活动场所。加强土地市场治理整顿，完善了土地收储制度，累计实现土地收益1亿元。住房公积金工作取得新进展，完成归集额1.1亿元。强化了城市管理，全市卫生状况、交通秩序、城市面貌有了新的改观。建成了长白一级公路，实现了乡乡通油路；实施了村村通等级公路工程，已完成418公里；通讯和信息设施不断完善。

　　——人民生活水平明显提高。2003年城镇居民人均可支配收入6424元，农民人均纯收入2525元，年均分别增长14%和18.2%。加强就业和社会保障工作，新开发就业岗位4.4万个，新增就业3.5万人；省级统筹养老保险、集体养老保险、失业保险覆盖率分别达到99%、93%和97%，均实现了100%发放；8.2万人纳入城市低保，19.6万农村贫困人口实现脱贫。全市基本实现了“三不欠”。与1998年相比，城乡居民人均储蓄存款由2087元增加到3307元；城镇人均居住面积由8.1平方米提高到14.5平方米，自来水普及率由75%提高到96%，人均公共绿地面积由4.7平方米增加到7.6平方米，人居环境和生活条件明显改善。

　　——各项社会事业全面发展。努力推进科技进步和创新，五年实施各级科技发展项目383项，取得各类科技成果135项、国家专利80项。大力发展教育事业，白城师范高等专科学校升格为师范学院，创办了4所大专院校，我市教育资源整合获省特别贡献奖；加快发展基础教育，高考进线率连续超过全省平均水平；推进素质教育和农村办学模式改革，义务教育水平有了新提高。教学条件明显改善，市区和县城新建教学楼（馆）33栋，农村中小学新建校舍、改造危房19万平方米，校舍砖瓦化率达100%，被评为全省校舍建设先进市。文化体育事业蓬勃发展，举办了文化节和体育那达慕大会等一系列大型文体活动，我市艺术团体获国家级奖3项，省级奖24项；在北京国际马拉松赛、全国第十届冬运会、省第十四届运动会等大型体育比赛中，我市运动员获金牌101枚；广播电视获省以上奖励176项，连续3年受到省表彰。建设了广电大厦、文化中心、疾控中心、中心血站、吉鹤和鹤城体育馆等一批高标准场馆，社会公益设施不断完善。深化医药卫生体制改革，医疗服务水平显著提高。全力组织防控非典阻击战，城乡联动，全民联防，团结奋战，夺取了抗击非典斗争的重大胜利，实现了把非典拒之白城之外的预期目标。计划生育工作在全省排序中位居前列，受到国家表彰。双拥工作取得成效，1999年被评为省双拥模范城，2003年跨入国家双拥模范城行列。全面完成了省政府确定的环境保护责任制目标，获省二等奖。地方志在全省率先完成了续志任务。民族、宗教、侨务、老龄、妇女儿童、残联、红十字会等事业有了新发展，国防后备力量建设、人防、防震减灾等工作取得了新成绩。

　　——精神文明和民主法制建设深入推进。广泛开展了“文明城市、文明村镇、文明行业”等群众性精神文明创建活动，强化社会公德、职业道德和家庭美德教育，城乡居民整体素质和文明程度明显提高。加强民主法制建设，认真执行人大决议和决定，定期向人大常委会报告工作，积极支持政协和各民主党派、工商联参政议政，邀请人大代表、政协委员视察重点工作进展情况。及时办理人大代表建议、议案和政协委员提案，五年共承办人大代表建议和议案148件、政协委员提案474件。建立健全社情民意反映制度、重大事项社会听证制度和专家咨询制度，推进了决策科学化、民主化。进一步完善了行政执法监督机制，认真组织行政执法检查，强化行政复议工作，依法行政和政府法制工作连续居全省前列。深入开展普法宣传教育和依法治市工作，干部群众的法律意识和依法办事的自觉性不断增强。加强群众来信来访工作，努力化解和妥善处理各种社会矛盾。全面落实社会治安综合治理各项措施，深入开展严打整治斗争和同“法轮功”等邪教组织的斗争，保持了政治安定和社会稳定。

　　——政府自身建设进一步加强。坚持“为民执政、科学理政、依法行政、从严治政”方针，努力转变观念、转变作风、转变职能，着力解决经济社会发展的重大问题。不断改善经济发展软环境，全面推行了直接办理制、窗口服务制、社会服务承诺制、全程服务制；加大行政审批制度改革力度，对行政审批项目进行全面清理、核查和审定，停止执行304项行政审批项目；市政务大厅已正式运行，对涉及39个部门和单位的256项行政审批服务事项实行集中办理，提高了办事效率。积极推进政务公开，在政府网站、广播电视、《白城日报》等媒体公布了部门审批事项和服务承诺；发挥市长和县（市、区）长公开电话作用，群众投诉办结率和满意率分别达96%和93%以上。认真落实“一岗双责”责任制，狠抓领导干部廉洁自律，纠正部门和行业不正之风，有效开展了对重点部门、重点资金、重点项目的经济监督和领导干部经济责任审计，推进了廉政建设。

　　过去的五年，我们始终坚持深入解放思想，不断更新观念，形成了人心思上思进、勇于开拓创新的浓厚氛围；始终坚持以经济建设为中心，凝聚全市各方面力量，克服前进中的困难，努力推进跨越式发展；始终坚持以改革为动力，注重解决经济社会发展中的主要矛盾和突出问题，推动各方面工作取得新突破；始终坚持“双招双引”基本市策和“四个走出去”策略，在更大范围、更广泛的领域推进对外开放，借助外力发展白城；始终坚持把维护稳定作为一项重要任务，正确处理改革、发展与稳定的关系，确保改革开放和经济建设顺利进行；始终坚持转变政府职能，加强依法行政，改进管理和服务方式，为经济社会发展创造良好环境。

　　五年来所取得的成绩，是全市各级干部和广大人民群众解放思想、开拓进取、团结奋斗的结果；是社会各界、方方面面大力支持的结果。我代表市政府向全市工人、农民、知识分子、各阶层群众和各级干部，向各级人大、政协、民主党派、工商联、人民团体及各界人士，向驻白部队、武警官兵、公安司法干警和中省直驻白单位，向对我们工作给予关心和帮助的离退休老领导、老同志表示衷心的感谢和崇高的敬意！

同时，我们也清醒地看到全市经济社会发展中存在的问题，主要是：经济总量小，结构不尽合理，市场机制不活；工业缺少大项目支撑，农民收入增长缓慢，现代服务业发展相对滞后，民营经济规模小；就业和社会保障压力增大，城乡部分弱势群体生活较困难，影响稳定的因素较多；政府职能转变还有差距，经济发展环境需要进一步改善。对这些问题，我们要高度重视，努力加以解决。

　　二、今后五年的目标和任务

　　今后五年，是推动白城二次创业、全面建设小康社会的关键时期，我们面临着难得的发展机遇和有利条件。国际经济进一步复苏，我国经济将进入一个新的快速增长期，振兴东北老工业基地和支持粮食主产区等战略的实施，全省二次创业热潮的兴起，为我们加快发展开辟了广阔空间；经过多年的建设，特别是近五年，白城进入了大开发、大建设、大发展的新时期，整体后发潜力大，已经初步具备了二次创业的基本条件。我们要抓住机遇，发挥优势，努力开拓创新，推进全民创业，全面振兴白城。

　　市政府工作的总体思路是：以邓小平理论和“三个代表”重要思想为指导，深入贯彻党的十六大和市委三届五次全会精神，认真落实全面、协调、可持续发展观。以加快全面建设小康社会进程为目标，用二次创业统领各方面工作，坚持开放带动、科教兴市、县域突破、人才兴业发展战略和更新观念争上游、负重前进加压力、改革创新找差距、跨越发展升位次工作方针。紧紧抓住振兴东北老工业基地的战略机遇，着力推进体制和机制创新，提高支柱产业规模化、传统产业现代化、新兴产业高新化、工业布局园区化水平，推动经济超常发展。加强政治文明和精神文明建设，促进社会全面进步，努力实现白城在全省二次创业中崛起。

　　全市经济社会发展的奋斗目标：到2008年，国内生产总值完成230亿元，年均增长13.3%；固定资产投资85亿元，年均增长13%；全口径财政收入17.6亿元，年均增长12.4%；城镇居民人均可支配收入9600元，年均增长8.4%；农民人均纯收入4000元，年均增长9.6%；人口自然增长率控制在6‰以内。民主法制建设更加完善；城乡居民思想道德、科学文化、健康素质进一步提高；生态环境明显改善，可持续发展能力不断增强，加快白城经济繁荣、科技进步、环境优美、社会文明、人民生活富足的步伐。

　　今后五年政府工作的主要任务：

　　第一，推进经济结构战略性调整，努力实现跨越式发展。立足白城实际，全面提升和优化第二产业，大力发展一、三产业，切实把经济工作的重点转到调整结构、提高增长质量和经济效益上来，促进全市经济协调快速发展。

　　建设六大产业基地，加快工业化步伐。抓住振兴东北老工业基地的战略机遇，发挥我市资源和产业优势，积极探索走新型工业化道路的有效途径，培育壮大支柱和优势产业，重点建设汽车零部件配套产业基地、纺织服装产业基地、现代中药及生物药产业基地、生态型特色农产品产业基地、能源产业基地、新型建材产业基地，增强综合经济实力。

　　着力发展现代农业，加快农业产业化步伐。坚持用工业化思维谋划农业和农村经济发展，进一步调整和优化结构，建设一批符合专业化、标准化、特色化和规模化要求的种植业、养殖业园区，依靠现代科技改造传统农业，积极发展优质、高产、高效、生态、安全的现代农业。培育壮大龙头企业，构筑农业产业化群体。加强农村社会化服务体系建设，发展多种形式的农业合作经济组织，提高农业和农村经济的组织化程度。

　　以现代服务业和旅游业为重点，加快第三产业发展步伐。大力发展信息、中介、金融、期货、证券、咨询、现代物流及会计、审计、律师等现代服务业，积极培育资本市场，高起点发展商业连锁经营，继续发展商贸、餐饮、仓储、交通运输等传统服务业及社区服务业。制定和完善旅游发展规划，加大投入，整合资源，加快景区景点建设，培育旅游企业，壮大旅游产业。

　　推动全民创业，加快民营经济发展步伐。强化全民创业意识，大力弘扬创业文化。建立健全民营经济政策支持体系，消除体制性障碍，在市场准入、投融资、土地使用、对外贸易等方面创造平等竞争的条件，依法保障民营企业的合法权益。支持中小民营企业加快发展，鼓励有条件的企业做大做强，推动民营经济实现大发展。

　　努力做大城市，加快城市经济发展步伐。按照“一市四区三个卫星城”长远发展框架，搞好城市建设总体规划设计，积极打造大城市布局板块。推进城市资产经营，重点抓好中心城市和县城的基础设施建设，提升城市承载能力。强化产业支撑，扩大经济总量，增强人口聚集、生产要素吸纳和综合服务功能，构筑新型城市经济格局。

　　第二，全面深化改革扩大开放，增强经济发展活力。认真贯彻《中共中央关于完善社会主义市场经济体制若干问题的决定》，推进各项改革。加快国有企业改革，稳步、规范实施企业“双退”，大力发展混合所有制经济。完善国有资产管理体制，建立归属清晰、权责明确、保护严格、流转顺畅的现代产权制度。完善公司法人治理结构，规范股东会、董事会、监事会和经营管理者的权责，建立和完善企业领导人员的激励约束机制。积极推进企业上市工作。进一步深化农村各项改革，加大解决“三农”问题的力度。抓好小城镇综合改革，培育经济强镇。同时推进其它方面的改革。

　　继续实施“四个走出去”策略，建立开放型经济体系。充分利用对内对外两个开放、筹集国内国际两类资金、拓展国内国际两个市场，以大开放促进大发展。加大“双招双引”力度，改进方式，创新手段，实施人才兴业战略和“千家客商、百家集团进白城”工程，提高引资引智实效。抓好外经外贸工作，着力培育外向型企业，开发外向型产品，创造条件扩大出口创汇。大力开展劳务输出，加快发展劳务经济。加强开发区建设，创新管理体制和发展机制，发挥其在对外开放和经济发展中的带动作用。

　　第三，加快项目和生态建设，积极推进可持续发展。我们要把加快白城发展的着力点集中到抓项目建设上来，以项目建设为载体，推进经济总量的扩张和综合实力的增强。重点围绕优势产业、资源开发、基础设施、社会事业和高新技术产业等组织项目建设，调动全市上下、方方面面的力量，投入到项目建设中来，形成千军万马跑项目、千辛万苦争取项目、千方百计上项目的局面。坚持以水利为核心，以绿化为重点，以管护为保证，以发展为目的，推进生态建设。继续抓好水利建设和造林绿化工程，合理利用自然资源，科学运作生态资本，积极发展绿色产业，不断改善生态环境。实施“瀚海桑田工程”，立足大规模开发、大产业牵动、高效益推进，培育壮大蚕业经济。

　　第四，切实维护群众利益，不断提高人民生活水平。坚持劳动者自主择业、市场调节就业、政府促进就业的方针，建立促进就业的组织领导体系、政策保障体系和社会服务体系。大力开发就业岗位，推行灵活多样的就业方式，创造更多的就业机会。积极培育劳动力市场，健全市场就业机制。努力提高中低收入者的收入水平。完善社会保障体系，保障困难群众的基本生活。推进城市扶困和农村扶贫开发，尽快使城乡贫困人口脱贫。强化公共服务设施建设，改善人民群众生活环境。加大住房公积金归集、信贷和管理力度，推动住房消费。建立和完善卫生服务、医疗保健体系，切实加强农村卫生工作，提高城乡居民的医疗保健水平。加快治安防控体系建设，维护社会稳定。

第五，加强政治文明和精神文明建设，促进社会全面进步。努力推进民主法制建设，完善向人大报告工作和向政协通报工作制度，认真办理人大代表建议、议案和政协委员提案，自觉接受人大依法监督和政协民主监督。密切同各民主党派、工商联和人民团体的联系。完善听证会、协调会等公民参与机制，畅通反映民意、集中民智的渠道，扩大人民群众对社会事务的知情权、参与权、监督权。以提高全体公民的基本法律素质为重点，广泛开展“四五”普法宣传教育。加强依法治市工作，积极开展法律服务。完善政府法制工作，全面推进依法行政。建立完善在源头上预防和治理腐败的制度措施，加强反腐败斗争和廉政建设。进一步推进社会主义精神文明建设，认真贯彻《公民道德建设实施纲要》，加强社会思想道德教育，广泛开展群众性精神文明创建活动，提高城乡文明程度。实施科教兴市战略，深化科技体制改革，加大科技成果引进、转化和推广力度，提高科技创新能力。坚持教育优先发展方针，推进教育体制改革，整合教育资源，构建现代国民教育和终身学习体系，建设学习型社会。加快文化、广播电视、体育、卫生等社会事业发展。

　　三、2004年的重点工作

　　2004年是新一届政府的开局之年，我们要深入贯彻省委、省政府和市委的部署，认真履行政府职责，不断解放思想，推进改革开放；突出项目建设，强化投资拉动；积极促进消费，努力扩大出口；优化经济结构，提高运行质量；改善发展环境，实行全民创业；统筹经济社会发展，加快白城二次创业步伐。

　　主要预期目标：全市国内生产总值完成141亿元，同比增长15%；固定资产投资53.5亿元，同比增长16%；全口径财政收入10.2亿元，按可比口径同比增长15%；城镇居民人均可支配收入7050元，同比增长9.7%；农民人均纯收入2750元，同比增长9%。重点抓好以下工作：

　　（一）培育壮大优势产业，着力提高工业效益。立足推进兴工富市，加快结构调整，扩大产业规模，提高工业运行质量。规模以上工业产值和利润同比分别增长15%和10%。突出优势产业发展重点，汽车配套和机械制造业尽快与国内外大集团、大公司并购重组，走合资合作、加快发展之路；纺织工业以抢占国际国内市场份额为主，实现生产规模的快速扩张，以规模优势营造产业优势；服装工业以提高地产面料成衣率为重点，创品牌、上档次，扩大市场覆盖面；医药工业围绕生物药、植化药、合成药的科技开发，加快具有自主知识产权的新药研发和生产；农产品加工业积极延伸产业链条，实施品牌化经营，加快发展龙头企业；建材业立足资源开发、新型材料推广应用，进一步提高生产能力。抓好企业提速增效工作，规模以上企业努力发挥市场前景好、生产潜力大的优势，不断扩大生产和销售；规模以下企业采取分工协作、配套加工等方式寻求新的发展。加大企业技术改造力度，全市技改投资完成7亿元，重点实施57个投资千万元以上的技改项目，抓好165种新产品和15种高新技术产品开发。组织企业走出去，宣传和推销优势、特色产品；推进电子商务、网上销售业务，积极拓展产品市场。

　　（二）加快发展生态效益农业，努力增加农民收入。充分利用国家扶持粮食主产区政策，加强粮食综合生产能力建设，多渠道增加农民收入。按照产业化布局规划建设好各类农业园区，着力推进农业增长方式转变。抓好万元田棚园区建设，扩大规模经营，提高品种质量，实现从量的扩张向提质增效转变，万元田棚发展到14.9万户。抓好牧业园区建设，积极发展优质奶牛、肉牛等地方特色养殖项目，搞好无规定动物疫病区建设。大力发展股份合作牧业经济，股份合作牧业户发展到2.5万户。抓好资源产品加工园区和农副产品贸易园区建设，相对集中地建设一批重点加工项目，积极开发绿色和无公害产品，打造特色品牌。加强农业标准化建设，推动农业科技进步。继续发展订单农业，强化合同规范和服务。组织好造林绿化，改善生态环境，完成造林30万亩，营造生态草50万亩、生态苇20万亩，加快发展林业股份经济。组织好“瀚海桑田工程”，完成植桑5万亩。加强水利建设，做好“引嫩入白”前期工作，实施“引洮入向”工程，抓好嫩江、洮儿河等堤防和重点水库除险加固、抗旱水源及节水灌溉、灌区续建配套工程建设，增强过境江河水、洪水蓄滞利用能力和人工增雨能力。强化国土资源管理和保护耕地工作。努力提高农业机械化水平，改善农业生产条件。

　　（三）大力发展商贸经济，增强辐射带动功能。围绕推进区域商贸中心城建设，加快培育和完善市场体系，重点完善洮南杂粮杂豆、大安白鹅、镇赉大牲畜、通榆关东马市、洮北红干椒等专业市场，建设具有地方特色、有规模、辐射力强的新型市场。加强市场管理，整顿市场秩序，维护经营者和消费者利益。积极发展现代流通业，引进国内外名企、名店、名品，推进超市、连锁经营、分销代理等现代流通方式。创造条件到省外、国外经商办店，开拓域外市场。社会消费品零售总额完成62.3亿元，同比增长10%。大力发展对外贸易，培育重点企业的自营出口能力，扩大纺织品、汽车零部件、农产品等出口规模。外贸进出口额完成5210万美元，同比增长15%。努力做好增收节支工作，积极培植财源，加强税收征管，严格控制支出，强化财政监督，提高资金使用效益。

　　（四）继续深化各项改革，推进体制和机制创新。组织好企业“双退”工作，坚持因企制宜，逐户研究企业退出方案，广泛宣传发动，严格按程序规范操作，力争今年全面完成“双退”任务。加快土地征用制度改革，切实落实最严格的耕地保护制度；坚持“依法、自愿、有偿”的原则，搞活土地使用权流转，推动土地规模经营；积极推进农村税费、粮食流通体制改革和农村信用社体制改革试点工作。抓好事业单位改革，逐步建立适应市场经济体制、充满生机与活力的管理体制和运行机制。搞好财政管理体制改革，健全公共财政体制。深化社会保障体制改革，组织好科技、教育、文化、卫生等方面改革，增强发展动力。

　　大力发展民营经济，动员广大干部群众领办、创办、兴办民营企业，全面落实发展民营经济的各项政策，积极扶持民营企业上规模、上水平，加快形成经济自主增长机制。全年新发展个体私营企业7500户，完成增加值和实缴税金同比分别增长20%和17%。

　　（五）突出抓好项目建设，拓展“双招双引”领域。认真研究国家、省有关政策和投资方向，积极谋划市场前景好、经济效益高、带动作用强的大项目、新项目。组织好2004年项目开发建设年的各项工作，千方百计抓投资、上项目，推进大发展。在优势产业项目上，重点抓好中一精锻连杆、轿车电线束、重型车变速箱、高档织物及高技术染色后整理、SMS特种纺织品、高档呢绒服装等项目建设，加快医药企业19条生产线GMP改造认证和国家级新药的引进开发；在资源开发项目上，大力实施林草业和蚕业开发、洮南向日葵精深加工和百万亩山杏种植及加工、大安白鹅和芝麻加工、镇赉黑豆油及系列食品、通榆木糖醇和蓖麻深加工、洮北专用玉米加工等项目。抓好风力发电和石油开发，开工建设洮北青山、洮南大通、通榆团结三个5万千瓦风电厂，创造条件加快石油开发。在基础设施项目上，启动实施白城热电厂扩建、三环路和垃圾处理等项目，搞好火电基地和煤炭化工项目规划；在高新技术产业项目上，继续抓好中国草原红牛生物胚胎工程建设，做好向日葵优良品种繁育、抗逆树种繁育等项目的争取工作，扩大项目建设成果。

　　抓住机遇，主动出击，到域外、国外有针对性地敲门招商；坚持以企业为主体与政府有效推动相结合，创新招商引资机制，增强实效性；采取“以商招商”、“以外引外”和网上招商等多样方式，吸引域外资本来我市投资，力争在引进新项目、大项目上取得新进展，在引进国内外大企业、大集团上实现新突破。全市招商引资完成44.4亿元。加快发展劳务经济，劳务输出30万人，实现收入10亿元。实施人才兴业战略，完善人才智力引进、流动、培育、使用机制，引进和培养我市急需的各类人才。

　　充分发挥开发区对外开放窗口作用，立足优势，突出特色，加快招商引资和项目建设步伐。白城经济开发区重点抓好工业园区建设，培育支柱产业；大安经济开发区重点抓好玉米秸秆综合开发等项目建设；查干浩特旅游经济开发区重点抓好景区设施完善和新的旅游项目开发；民营经济开发区重点抓好绿色食品、甘草系列加工等项目建设；科尔沁精品牧业发展区重点抓好优质奶牛、肉牛规模养殖；洮南农副产品加工贸易园区重点抓好农特产品加工销售。

　　（六）抓好基础设施建设，不断改善城乡面貌。强化经营城市意识，坚持高起点规划、高质量建设、高效能管理，继续在全市范围内开展城市开发建设管理总体战。全市计划投资35.5亿元，其中市区投资17.5亿元，各县（市）投资18亿元。市区集中抓好十四项工程（124件实事），即以游园、绿地、护城河等为重点的城市环境建设工程，以保胜路、和平街等新建改造及人行道铺装为重点的道路建设工程，以金辉互通立交桥、污水处理、供热等为重点的基础设施建设工程，以扩大供水范围、封闭自备水源井为重点的城市供水工程，以经济适用住房和廉租房为重点的住宅建设工程，以加快旧城改造为重点的行政新区建设工程的规划运作，以及亮化、蓝天、环卫设施、社会公益事业建设和治理“十乱”等工程。各县（市）实施四十项工程，大安市完成道路、排水、燃气等工程；洮南市完成住宅、绿化、垃圾处理等工程；镇赉县完成广场、亮化、环卫设施建设等工程；通榆县完成供水、供热、标准街路等工程。

　　按照全市旅游总体规划，大力开发生态旅游业。继续抓好向海、莫莫格、查干浩特、环市区等景区景点建设和完善，积极开发具有白城特色的旅游产品，提高行业管理和服务水平，促进旅游业加快发展。

　　组织好公路建设，加快实施村村通等级公路工程。继续抓好信息、通讯等设施建设，大力发展信息产业。

　　（七）积极发展各项社会事业，切实维护社会稳定。加快科技创新，抓好科技发展项目和科技示范园区建设，推进优势产业和重点企业开发一批具有自主知识产权的关键技术和名牌产品，提高科技对经济发展的贡献率。调整教育结构，巩固提高义务教育，积极发展高中教育和职业教育，不断完善高等教育。大力发展文化体育事业，组织开展丰富多彩的群众性文体活动，加强社区和农村文化建设，倡导文明风尚。广泛开展爱国卫生运动，努力创建卫生城。坚持不懈地抓好非典、禽流感等传染性疾病的防控，建立健全突发公共卫生事件应急机制，实行严格的责任制，把防控措施落到实处。抓好计划生育工作，确保完成全年人口计划。充分发挥广播电视宣传窗口作用，提高宣传质量，营造良好的舆论氛围。进一步加强国防后备力量建设，开展好双拥共建活动。认真做好民族、宗教、侨务、地方志、防震减灾、气象、人工降雨、人防、老龄、妇女儿童、残联、红十字会等工作，促进社会事业全面发展。

　　认真落实各项就业政策，多渠道开发就业岗位，拓宽就业领域。鼓励和扶持下岗失业人员自谋职业、自主创业。加强社区服务组织建设，搞好就业指导服务。强化培训，提高劳动者就业技能。加大养老、失业保险扩面、基金征缴和清欠力度，确保及时足额发放。做好城镇居民最低生活保障工作，大力发展社会救济、社会福利、优抚安置和社会救助事业，增强保障功能。努力做好人民群众来信来访工作，严格实行首访负责制、领导包案制、书面答复制和责任追究制，妥善处理涉及群众切身利益的矛盾和问题。强化社会治安综合治理，深入开展严打整治斗争，坚决打击“法轮功”等邪教组织的破坏活动。抓好安全生产和消防、交通等安全管理，防止重大事故发生。

　　（八）进一步转变政府职能，营造良好的发展环境。坚持“四政” 方针，加强和改进政府工作，努力把政府经济管理职能转到主要为市场主体服务和创造良好发展环境上来。

　　深入解放思想，保持与时俱进的精神状态。面对新的形势和任务，我们要在思想观念上有一个大的转变。按照思想和工作体现时代性、把握规律性、富于创造性的要求，坚决破除不利于全民创业的思想观念，增强抢抓机遇意识和主动招商积极创业意识，以观念更新促进工作创新。广泛开展“解放思想、全民创业”主题教育活动，充分调动广大干部群众的积极性和创造性，形成全民创业的浓厚氛围。

　　加强政风建设，强化软环境治理。认真贯彻《行政许可法》，深化行政审批改革，全面清理行政许可项目，规范审批主体和行为。充分发挥政务大厅作用，完善运行机制，简化和规范办事程序，公开办事制度，提高服务质量和效率。建立健全环境建设长效机制，实行优化环境的目标责任制和损害环境的责任追究制，使环境建设制度化、规范化。加强市长和县（市、区）长公开电话工作，切实为民排忧解难。大力整顿社会信用环境，推进社会信用体系建设。加快政务信息化建设，以政务信息化带动促进企业和社会信息化。

　　切实转变作风，狠抓工作落实。坚持深入实际调查研究，密切同人民群众的联系，深怀爱民之心，恪守为民之责，善谋富民之策，多办利民之事，把人民群众的利益维护好、实现好、发展好。坚决克服官僚主义、形式主义，努力解决工作中的实际问题。增强全局观念，做到局部利益服从整体利益，确保政令畅通。倡导雷厉风行、求真务实的工作作风，建立明确的岗位责任制，凡是确定的工作任务，要一抓到底，真正落到实处。全面落实廉政建设责任制，建立健全反腐倡廉制度体系，坚决纠正群众反映强烈的不正之风，努力从源头上预防和治理腐败。强化审计监督，维护经济秩序，促进廉政建设，保障经济社会健康发展。

　　各位代表，振兴东北老工业基地的历史机遇千载难逢，二次创业的热潮正在兴起。形势催人奋进，使命光荣艰巨。我们要在市委领导下，深入贯彻“三个代表”重要思想，与时俱进，开拓创新，扎实工作，为加快白城全面建设小康社会进程而努力奋斗！
